# Influence of the diagnosis chronic rhinosinusitis on asthma response to omalizumab in severe, uncontrolled asthmatics

**DOI:** 10.1186/2045-7022-5-S4-O11

**Published:** 2015-06-26

**Authors:** Ina Sintobin, Maria del Carmen Vennera, Joaquim Mullol, Claus Bachert

**Affiliations:** 1 Upper Airway Research Laboratory, Ghent University, Ghent, Belgium; 2 Hospital Clínico de Barcelona, Institut Clínic del Tòrax, Servei de Pneumologia i Allèrgia Respiratòria, Barcelona, Spain; 3 University of Barcelona, Institut d’Investigacions Biomèdiques August Pi i, Barcelona, Spain; 4 Ghent University, Upper Airway Research Laboratory, Ghent, Belgium

## Background

Omalizumab is a humanized anti-immunoglobulin E (IgE) monoclonal antibody that has been approved as add-on therapy for the treatment of adults with moderate-to-severe (United States) or severe (Europe) allergic asthma, inadequately controlled after treatment with high-dose inhaled corticosteroids plus long-acting β-agonists. The clinical efficacy of Omalizumab has not only been shown in the treatment of severe uncontrolled asthma, but also in the treatment of nasal polyposis and comorbid asthma. The aim of this analysis was to examine whether the diagnosis of chronic rhinosinusitis (CRS) influenced the asthma response to Omalizumab treatment.

## Methods

This study retrospectively analysed data from 70 patients with severe, uncontrolled asthma treated with Omalizumab. A skin prick test (SPT) was performed in all patients. In serum, specific Immunoglobulin E (IgE) to Staphylococcal enterotoxins (SE) and total IgE were measured by Immunocap. Asthma response was evaluated by physician’s Global Evaluation of Treatment Effectiveness (GETE-score); GETE 0, 1 and 2 were considered as non-responders, GETE 3 and 4 as responders.

## Results

The mean age of the patients was 54.4 years and 62% of them were female. 84.3% of the asthmatics responded to Omalizumab treatment. Within the total patient group, 55.7% had comorbid nasal polyps and 83.8% had antibodies to staphylococcal enterotoxins in their serum. Within the SE-IgE positive group, 54.4% had an excellent GETE score, compared to 30.4% with good score and 14.3% with moderate GETE-score. A higher SE-IgE concentration was found in patients with CRS with nasal polyps (CRSwNP) and a strong correlation between SE-IgE and total IgE was observed. The proportion of responders tended to be higher in the group diagnosed with CRSwNP compared to CRS without nasal polyps (CRSsNP), and higher in CRSsNP compared to no CRS (89.7% vs. 85.7% vs. 81.8%); the differences were not statistically significant. The presence of CRS thus did not reduce the overall high response rate to Omalizumab.

## Conclusions

We here showed that a high proportion of severe uncontrolled asthma patients are sensitized to staphylococcal enterotoxins and more than half of these severe asthmatics have comorbid nasal polyposis. There is a trend towards a better asthma response in patients with antibodies to SE and in patients with CRSwNP.

